# Kostenfaktor „ambulante Wundversorgung“ in der Notaufnahme: Kosten-Erlös-Defizit der ambulanten Wundversorgung in einer deutschen Universitätsklinik

**DOI:** 10.1007/s00113-020-00819-1

**Published:** 2020-05-12

**Authors:** Cora R. Schindler, Thomas Lustenberger, Ingo Marzi, René D. Verboket

**Affiliations:** Klinik für Unfall‑, Hand- und Wiederherstellungschirurgie, Goethe-Universität Frankfurt, Theodor-Stern-Kai 7, 60590 Frankfurt am Main, Deutschland

**Keywords:** Wundversorgung, Notaufnahme, EBM, Vergütung, Gesundheitsökonomie, Wound care, EBM, Emergency room, Health economics, Remuneration

## Abstract

**Hintergrund:**

Die Erstversorgung von Wunden und kleinere chirurgische Eingriffe gehören neben der hochspezialisierten Medizin zu den allgemein notwendigen Grundleistungen der Notfallversorgung in den Kliniken. Die Vergütung der ambulanten Notfallleistungen für gesetzlich Versicherte erfolgt derzeit nach dem Einheitlichen Bewertungsmaßstab (EBM), welchem die betriebswirtschaftliche Aufwandserfassung des niedergelassenen Sektors als Kalkulationsgrundlage dient. Krankenhäuser haben im Vergleich zu Arztpraxen wesentlich höhere Vorhaltungskosten.

**Ziel der Arbeit:**

In dieser Arbeit wird das entstehende Kosten-Erlös-Verhältnis der ambulanten Wundversorgung in einer Notaufnahme durch die Vergütung nach EBM analysiert.

**Material und Methoden:**

Die Daten wurden in der Notaufnahme des Universitätsklinikums Frankfurt am Main über 12 Monate erhoben. Eingeschlossen wurden alle Patienten, die in diesem Zeitraum eine Wundversorgung mittels Naht erhielten. Die Kosten wurden der Abrechnung nach EBM 01210 (bzw. 01212) mit der Zusatzpauschale für kleinchirurgische Eingriffe EBM 02301 gegenübergestellt.

**Ergebnisse:**

Im Beobachtungszeitraum wurden 1548 Patienten versorgt; das entspricht 19,52 % aller unfallchirurgischen Fälle. Den Kosten einer Standardwundversorgung in Höhe von 45,40 € steht eine Vergütung von 31,83 € gegenüber. Die Berechnung des Gesamterlöses weist einen Defizitbetrag von 13,57 € pro ambulantem Fall auf; dies entspricht einem Jahresdefizit von 21.006,36 €.

**Diskussion:**

Es konnte gezeigt werden, dass ohne Betrachtung der relevanten Vorhaltekosten in keinem Fall eine Kostendeckung erreicht werden kann.

Die bisherige Vergütung der ambulanten Wundversorgung nach EBM erscheint unzureichend. Eine Anpassung bzw. Zusatzvergütung scheint notwendig, um eine ausreichende Versorgungsqualität in Zukunft sicherstellen zu können.

## Abstract (en)

**Background:**

In addition to highly specialized medicine, the initial treatment of wounds and minor surgical interventions are generally necessary basic services of emergency care in hospitals. The reimbursement of outpatient emergency services for persons with statutory insurance is currently based on the uniform assessment standard (EBM), where the recording of business expenses in the private practice sector serves as the basis for the calculation. Hospitals have considerably higher maintenance costs than medical practices.

**Objective:**

In this article the resulting cost-revenue ratio of outpatient wound care in an emergency department is analyzed through the reimbursement according to EBM.

**Material and methods:**

The data were collected in the emergency surgical department of the University Hospital Frankfurt am Main over 12 months. Included were all patients who received sutured wound care during this period. The costs incurred were compared to the remuneration according to EBM 01210 (or 01212) with the additional flat rate for small surgical procedures EBM 02301.

**Results:**

During the observation period 1548 patients were treated, i.e. 19.52% of all trauma surgery cases. The resulting costs of a standard wound care of 45.40 € are offset by a remuneration of 31.83 €. The calculation of the total revenue shows a deficit amount of 13.57 € per outpatient case, this corresponds to an annual deficit of 21,006.36 €.

**Conclusion:**

It could be shown that even without consideration of the relevant holding costs, cost coverage cannot be achieved in any case.

The previous reimbursement of outpatient wound care on the basis of the EBM appears to be inadequate. In the future, an adjustment or supplementary remuneration seems to be necessary in order to ensure sufficient quality of care.

## Einleitung

Jährlich werden bundesweit ca. 18,6 % der gesetzlich Krankenversicherten (GKV) in der ambulanten Notfallversorgung behandelt. Das entspricht rund 7,5 Mio. Menschen [17]. Davon stellten sich 2017 laut Statistischem Bundesamt (DESTATIS) 1,99 Mio. Verletzte in den Notaufnahmen der Krankhäuser vor. Nach § 75 Abs. 1 Satz 2 SGB V sind die kassenärztlichen Bundesvereinigungen (KBV) zur Sicherstellung eines Notdienstes verpflichtet, wobei die Versicherten frei wählen können, welche Form des Notdienstes sie in Anspruch nehmen [7, 23]. Die Gesundheitskosten in Deutschland beanspruchen 11,5 % des Bruttoinlandsproduktes. Dabei entstehen bundesweit jedes Jahr Krankhauskosten in Höhe von 105,7 Mrd. € [24, 25]. Von diesen Fällen werden 80 % gegenüber der GKV und 20 % gegenüber Berufsgenossenschaften oder privatärztlich abgerechnet. Die Vergütung der ambulanten Notfallleistungen für gesetzlich Versicherte erfolgt nach dem Einheitlichen Bewertungsmaßstab (EBM). Der EBM regelt gemäß § 87 Abs. 2 SGB V Inhalt und Umfang abrechnungsfähiger Leistungen im Bereich der vertragsärztlichen Versorgung in einem Punktesystem, welches nach regionalen Gebührenordnungen in Euro-Preise umgerechnet wird. Kalkulationsgrundlage ist die betriebswirtschaftliche Aufwandserfassung im niedergelassenen Bereich [3, 6, 10]. Krankenhäuser haben jedoch im Vergleich zur Arztpraxis wesentlich höhere Vorhaltungskosten. In den Notaufnahmen entfallen rund ein Drittel der Kosten auf den ärztlichen Dienst, ein Drittel auf Pflege- bzw. Funktionsdienste, 9 % auf medizinische Sachkosten und 25 % auf Infrastrukturkosten, wie Gebäudebewirtschaftung, Verwaltung, Zentralsterilisation, etc. [21, 26]. Die pro vorstelligem Patienten erhobene Notfallpauschale EBM 01210 (bzw. 01212) sieht einen obligaten, persönlichen Arzt-Patient-Kontakt mit weiteren fakultativen Bestandteilen, wie die Erhebung des funktionellen Ganzkörperstatus und Erstellung des Arztbriefs, vor. Die Pauschale kann unabhängig vom Vorstellungsgrund nur einmal im Quartal pro Patient im selben Krankenhaus und mit derselben Versicherung abgerechnet werden [7, 12]. Nur bestimmte Einzelleistungen, wie die Wundversorgung mittels Naht (EBM 02301), werden zusätzlich erstattet [13]. Die Erstversorgung von Wunden sowie die Durchführung kleinerer chirurgischer Eingriffe gehören neben der hochspezialisierten Medizin weiterhin zu den allgemein notwendigen Grundleistungen in der Notfallversorgung der Kliniken. Entsprechend der aktuellen S1-Leitlinie müssen akute Wunden in einem Zeitfenster unter 6 h versorgt werden [16]. Zur Behandlung gehören neben der chirurgischen Wundversorgung eine eingehende Anamneseerhebung und Untersuchung mit Ausschluss von Begleitverletzungen, die Prüfung von Sensibilität, Perfusion, Motorik sowie die sorgfältige Dokumentation und ergänzende radiologische Bildgebung bei V. a. eine knöcherne Begleitverletzung. Ebenso gehören die vorherige Infiltration mit einem Lokalanästhetikum und die Wundreinigung und Desinfektion dazu. Liegt außerdem kein ausreichender Tetanusimpfschutz vor, wird nach den Empfehlungen der STIKO eine Auffrischung oder Simultanimpfung durchgeführt [16]. Oft führt die unzureichende Vergütung der Notfallleistungen zu einer regelhaften Kostenunterdeckung in den Notaufnahmen [17, 22].

Ziel dieser Arbeit ist es, das Kosten-Erlös-Verhältnis der ambulanten Wundversorgung in einer universitären Notaufnahme anhand der Vergütung von Notfallleistungen auf der Kalkulationsgrundlage des EBM zu untersuchen. Es soll evaluiert werden, ob die im EBM gegebene Abrechnungsgrundlage mit den auflaufenden Kosten im Einklang steht.

## Studiendesign und Methoden

### Studiendesign und Datenakquise

Mittels einer systematischen Abfrage im Krankenhausinformationssystem (Hospital Information System, HIS) wurden alle Patienten identifiziert, die im Zeitraum von Juni 2018 bis Juni 2019 in der Zentralen Notaufnahme (ZNA) der Abteilung für Unfall‑, Hand- und Wiederherstellungschirurgie am Universitätsklinikum Frankfurt am Main eine Wundversorgung mittels Naht (EBM 02301) erhielten. Fälle mit unklarer Art der Wundversorgung oder unzureichender Dokumentation sowie stationär aufgenommene Patienten wurden ausgeschlossen. Insgesamt konnten so 1548 Fälle identifiziert werden. Es wurden keine personenbezogenen Daten erhoben. Die Patientenzahl enthält ausschließlich gesetzlich Versicherte. Selbstzahler, Privatpatienten und berufsgenossenschaftliche Fälle wurden aufgrund anderer Abrechnungsmodalitäten ausgeschlossen. Die vorliegende Studie folgt den STROBE-Richtlinien für Beobachtungsstudien (Strengthening The Reporting of Observational Studies in Epidemiology) sowie den RECORD-Richtlinien für Observationsstudien (Reporting of studies Conducted using Observational Routinely-collected Data) [4, 8].

### Vergütungspauschalen

Die Erlöse wurden anhand der Notfallpauschale EBM 01210 im organisierten Notdienst bei Inanspruchnahme von Montag bis Freitag zwischen 07:00 und 19:00 Uhr und der Notfallpauschale EBM 01212 zwischen 19:00 und 07:00 Uhr des Folgetages, ganztägig an Samstagen, Sonntagen, gesetzlichen Feiertagen und am 24. und 31. Dezember berechnet [14, 15]. Die Wundversorgung wurde zusätzlich mit der Pauschale EBM 02301 „Kleinchirurgischer Eingriff II und/oder primäre Wundversorgung mittels Naht“ berechnet, welche neben der Hautnaht die Entfernung eines unter der Hautoberfläche gelegenen Fremdkörpers, die Deckung eines Hautdefekts sowie die Eröffnung eines subkutanen Panaritiums oder Abszesses beinhaltet [13].

### Kostenkalkulation

Zur Fallkostenkalkulation wurde in Zusammenarbeit mit dem operativen Controlling eine Aufschlüsselung der anfallenden Einzelposten erstellt. Zur Berechnung der Personalkosten wurde der Standardfall (ein Arzt und eine Pflegekraft an der Behandlung beteiligt) zugrunde gelegt und das jeweilige Personal nach ihren spezifischen Entgelten betrachtet. Über 3 Monate wurde die Behandlungsdauer der Wundversorgungen von ärztlicher und pflegerischer Seite dokumentiert und so jeweils ein Mittelwert erstellt (ärztlich: 17,87 min, pflegerisch: 32,08 min). Die Minutenkosten wurden auf Basis der ab 20.02.2018 geltenden Entgelttabelle nach dem Tarifvertrag für Ärztinnen und Ärzte an den hessischen Universitätskliniken (TV-Ärzte Hessen, [18]) berechnet. Bei einer 42-h-Woche wurden für den unfallchirurgischen Notaufnahmedienst (Durchschnittsgehalt aus 1./4./7. Weiterbildungsjahr) die Minutenentgelte errechnet (Tab. 1). Der Arbeitgeberanteil für die Lohnnebenkosten im Jahr 2018 wurde mit 19,375 % berechnet. Hierzu wurden die Renten‑, Arbeitslosen‑, Kranken- und Pflegeversicherung für einen beispielhaften, kinderlosen Arbeitnehmer mit Lohnsteuerklasse 1 und gesetzlicher Krankenversicherung zugrunde gelegt. Die Nettojahresarbeitsstunden wurden unter der Berücksichtigung einer Ausfallzeit von 2,1 % für Ärzte berechnet ([11]; Tab. 2). Der Minutenpreis des Pflegepersonals wurde anhand des Tarifvertrags für die Johann-Wolfgang-Goethe-Universität Frankfurt am Main (TV-G-U) in der Fassung des Änderungstarifvertrages vom 11.09.2017 (Entgelttabelle für Pflegekräfte, gültig ab 01.01.2018) für 38,5 Wochenstunden ermittelt. Als Referenz diente die vorwiegend beteiligte Entgeltgruppe P8. Die durchschnittlichen Personalkosten für die Entwicklungsstufen 3 und 4 wurden gemittelt. Der Arbeitgeberanteil der Lohnnebenkosten wurde entsprechend der Ärzte mit 19,375 % berechnet und eine Ausfallzeit von 6,85 % berücksichtigt [11]. Zur Berechnung der Kosten für Verbrauchsmaterial, Desinfektionsmittel und Lokalanästhetikum wurden die Einkaufspreise über den zentralen Einkauf bzw. der Klinikapotheke zugrunde gelegt. Die Wundversorgung wurde für alle Patienten entsprechend der S1-Leitlinie durchgeführt und die zuvor ermittelte Behandlungsdauer einer Standardwundversorgung zugrunde gelegt.

**Table 1:** 

	Artikel	Anzahl	Kosten (in €)
*Verbrauchsmaterial*	Wundversorgungset, steril^a^	1	9,30
	Tupfer, steril (3er-Pack)	1	0,21
	Abdecktuch, steril	1	0,46
	Lochtuch, steril	1	0,57
	Haube	1	0,05
	Mundschutz	2	0,07
	Handschuhe, steril	1	0,31
	Nahtmaterial	1	3,91
	Einmalskalpell, steril	1	3,50
	Spritze, 10 ml, steril	2	0,08
	Kanüle, steril	2	0,02
	Kompressen, steril	2	0,06
	Mullbinde	1	0,07
	NaCl, 0,9 %ig, 10 ml	2	0,06
	Desinfektion	300 ml	1,78
	Lokalanästhetikum	5 ml	0,29
	*Zwischensumme*	*20,74*	
*Unfallchirurgie, Arzt*	Durchschnittliche Dauer der Aufnahme und Wundversorgung in Minuten	17,87	*11,15*
*Unfallchirurgie, Pflege*	Durchschnittliche Dauer der Triage, Vorbereitung, Assistenz und Nachbereitung in Minuten	32,08	*13,51*
*Gesamtkosten*			*45,40*

^a^Einmalset, inkl. Schere, Pinzette, Nadelhalter, Nierenschale, Becher, 5 kleine Kompressen

**Table 2:** 

**Arzt, Unfallchirurgie**	**–**	**Jahresgehalt**	**Inkl. Arbeitgeberanteil**
*–*	1. Jahr	56.970,48 €	68.008,51 €
	4. Jahr	67.401,78 €	80.460,87 €
	7. Jahr	69.209,94 €	82.619,67 €
*Mittelwert*			*77.029,68* *€*
*Bruttojahresstunden*	365 Tage − 115 Tage (Wochenenden und Feiertage in Hessen 2018) = *250 Tage*		
	42 h /5 Tage = 8,4 h pro Tag Arbeitszeit 250 Tage ⋅ 8,4 h = *2100 Arbeitsstunden pro Jahr*		
*Nettojahresstunden*	2100 h pro Jahr − 2,1 % Ausfallzeit = 2055,9 h = *123.354* *min*		
*Minutenentgelt*	77.029,68 € /123.354 min = *0,624* *€ pro Minute*		

Die Kosten der einzelnen Faktoren wurden zu einem Gesamtkostenbetrag addiert und anhand der prozentualen Fallverteilung auf die jeweilige EBM-Pauschale mit der Gesamtvergütung verglichen (Tab. 3).

**Table 3:** 

**Kosten**			
Personalkosten			24,66 €
Materialkosten			20,74 €
*Summe*			*45,40* *€*
**Erlöse**			
EBM 02301 nach GOÄ Hessen	13,96 €	100 %	–
EBM 01210 nach GOÄ Hessen	12,99 €	37,21 %	–
EBM 01212 nach GOÄ Hessen	21,10 €	62,79 %	–
(EBM 01210 * 564 + 01212 * 972) / 1548		Ø	–
*Summe*			*31,83* *€*
*Gesamterlös pro Fall*			***−13,57*** ***€***
*Kosten-Erlös-Verhältnis pro Jahr*			***−21.006,36*** ***€***

## Ergebnisse

In der Zeit vom Juni 2018 bis Juni 2019 stellten sich 1548 Patienten mit Wunden in der Notaufnahme vor, welche ambulant mittels Hautnaht versorgt wurden. Das entspricht 18,77 % aller Patienten, die in der unfallchirurgischen Notaufnahme in diesem Zeitraum versorgt wurden. Von diesen Patienten stellten sich 37,21 % (564 Fälle) in der Zeit von Montag bis Freitag zwischen 7:00 und 19:00 Uhr (EBM 01210) vor und wurden mit 120 Punkten, d. h. 12,99 € nach der Gebührenverordnung für Ärzte (GOÄ) in Hessen abgerechnet. Die Übrigen 62,79 % (972 Fälle) wurden entweder nachts oder am Wochenende behandelt (EBM 01212) und mit 195 Punkten, entsprechend 21,10 € (GOÄ Hessen), bewertet. Für die Durchführung einer Wundversorgung wurden nach EBM 02310 13,96 € (129 Punkte, GOÄ Hessen) abgerechnet.

### Erlöse

Es ergibt sich ein Gesamterlös von 26,95 € für eine Wundversorgung tagsüber unter der Woche und 35,06 € pro nächtlichem Notfall bzw. am Wochenende oder an Feiertagen. Hochgerechnet auf die ermittelten Fallzahlen von 564 Fällen nach EBM 01210 entspricht das 15.199,80 € für Wundversorgungen unter der Woche. Die 972 nachts und am Wochenende (EBM 01212) durchgeführten Behandlungen erzielten Einnahmen von 34.078,32 €. Somit errechnet sich für den Zeitraum vom Juni 2018 bis Juni 2019 (12 Monate) ein Gesamterlös von 49.278,12 €. Das entspricht 12.319,53 € pro Quartal und durchschnittlich 31,83 € pro Fall für die ambulante Wundversorgung in der Notaufnahme.

### Kosten

Die Kostenkalkulation für Personal erfolgt, wie im Abschn. „Material und Methoden“ beschrieben und in Tab. 1 aufgeführt. Für die Personalkosten der Ärzte im Dienst wurden 0,624 € pro Minute berechnet. Für das Pflegepersonal wurde ein Minutenentgelt von 0,412 € berücksichtigt. Der durchschnittliche Zeitaufwand des ärztlichen Personals für eine Wundversorgung, inklusive der eingehenden Anamnese, Untersuchung, Beratung bzw. Aufklärung, Behandlung, sowie die abschließende Erstellung eines Arztbriefs und das Ausstellen von Rezepten und Krankmeldung belaufen sich auf durchschnittlich 17,87 min. Die assistierende Pflegekraft hat darüber hinaus einen Mehrzeitaufwand von 14,21 min durch die vorherige Triage, Patienten- und Materialvorbereitung sowie Verband und Nachbereitung des Behandlungsraumes und ist damit insgesamt 32,08 min mit der Versorgung des Patienten beschäftigt. Damit entstehen 24,66 € Gesamtpersonalkosten, anteilig 11,15 € für die ärztliche und 13,51 € für die pflegerische Leistung.

Die Verbrauchsmaterialien kosten pro Wundversorgung 20,74 € (Tab. 2). Kosten für Reinigung, Verwaltung und Geräte- und andere Vorhaltekosten sind nicht berechnet.

### Kosten-Erlös-Verhältnis

Es entstehen pro versorgtem Patienten Gesamtkosten von 45,40 €. Dem gegenübergestellt wurde die durchschnittliche Vergütung von 31,83 € pro Fall.

Nach Abzug der Kosten von den Erlösen gegenüber der Krankkassen ergibt sich ein Defizit von 13,57 € pro Fall. Dies entspricht einem Jahresdefizit von 21.006,36 € bzw. 5251,59 € im Quartal für die ambulante Wundversorgung in der Notaufnahme (Tab. 3).

## Diskussion

Ziel dieser Arbeit ist es, Kosten und Erlöse der ambulanten Wundversorgung an einer deutschen Universitätsklinik als Beispiel eines Maximalversorgers gegenüberzustellen. Die Wundversorgung wurde gewählt, um einen häufigen (18,77 %, vgl. oben) ambulanten Behandlungsfall und dessen Vergütung nach EBM abzubilden.

Nach §108 SGB V sind zugelassene Krankenhäuser zur Teilnahme an der Notfallversorgung verpflichtet. Herausforderung ist hierbei die Gewährleistung einer erreichbaren, hochwertigen, aber auch wirtschaftlichen Notfallversorgung. Dem gegenüber stehen die zunehmend schwierigere Organisation und Finanzierbarkeit durch die von Patienten selbst gesteuerte Inanspruchnahme von Notfallleistungen [9, 19, 28]. Seit Jahren wird das Defizit zwischen zu hohen Kosten und einer zu geringen Vergütung in deutschen Notaufnahmen thematisiert. Ein erster Ansatz, Gründe für diese Vergütungsdefizite offenzulegen, ist, einzelne Leistungen anhand ihrer Wirtschaftlichkeit zu analysieren [10]. Hier wurde durch mehrere Autoren bereits die generelle Frage aufgeworfen, ob eine ambulante Versorgung von Patienten über die Notaufnahme überhaupt noch wirtschaftlich sinnvoll ist, da v. a. Maximalversorger Probleme mit der Finanzierung haben. Die bisher durchgeführten Untersuchungen konzentrierten sich v. a. auf die Polytraumaversorgung, besondere Krankheitsbilder, oder bildeten die Gesamtkosten der ambulanten Notfallversorgung ab. Diese errechneten einen fallbezogenen Erlös von 32 € bei durchschnittlichen Kosten von 120 € in der ambulanten Notfallversorgung. Daraus resultierte ein Fehlbetrag von 88 € pro Fall [9, 19, 28, 29, 31, 33, 34]. Anhand dieser Arbeit konnte am Beispiel einer Wundversorgung, die in jeder Klinik täglich mehrfach durchgeführt wird, ebenfalls ein Vergütungsdefizit gezeigt werden, hier von 13,57 € pro Fall.

Knapp 2 Mio. Verletzte stellen sich jährlich in den Notaufnahmen der öffentlichen Krankhäuser vor [25]. Rund die Hälfte (52 %) aller Notfallpatienten wird im Krankenhaus nur ambulant behandelt, davon werden 80 % mit der GKV über das EBM-System abgerechnet. Während die Anzahl an Notfällen im kassenärztlichen Bereitschaftsdienst um 15 % von 12,3 Mio. (2009) auf 10,5 Mio. (2015) sank, stieg gleichzeitig die Zahl der ambulanten Notfälle im Krankenhaus von 6 Mio. in 2009 auf über 8,5 Mio. im Jahr 2015 [21]. Dies entspricht einem Zuwachs um 42 % und zeigt die deutliche Verschiebung der Notfallvorstellung in die Notaufnahme [3, 22]. Für die Krankenhäuser gewinnt so die Versorgung in der Notaufnahme eine immer größere Bedeutung [30]. Eine Vorstellung zur Wundversorgung in der Notaufnahme erfolgte im ausgewerteten Jahr durch 1548 Patienten; dies entspricht 18,77 % aller unfallchirurgisch, ambulant behandelten Fälle. Davon stellten sich 37,21 % der Patienten von Montag bis Freitag zwischen 7:00 und 19:00 Uhr, 62,79 % entweder nachts oder am Wochenende vor. Im Projektbericht zur Notfallversorgung in Deutschland vom April 2018 dokumentiert die Kassenärztliche Bundesvereinigung, dass die Anzahl der ambulanten Notfälle am Wochenende um rund 39 % steigt. Die meisten ambulanten Notfallbehandlungen werden werktags in der Zeit zwischen 18:00 und 20:00 Uhr durchgeführt, am Wochenende zwischen 10:00 und 12:00 Uhr [21]. Die Nachfrage ist somit erwartungsgemäß außerhalb der regulären Öffnungszeiten der niedergelassenen Ärzte am höchsten.

Der EBM regelt Inhalt und Umfang anrechnungsfähiger Leistungen im Bereich der vertragsärztlichen Versorgung. Kalkulationsgrundlage ist die betriebswirtschaftliche Aufwandserfassung im niedergelassenen Bereich [6]. Eine Übertragbarkeit auf Krankenhäuser ist zweifelhaft, da die Kostenstrukturen in keiner Weise vergleichbar sind. Für den Standardfall der ambulanten Wundversorgung mittels Naht konnte durch die EBM-Pauschalen keine Kostendeckung erreicht werden. Es errechnet sich für 1548 behandelte Patienten ein relevanter Fehlbetrag von 21.006,36 € im Jahr. Aus ökonomischer Sicht ist somit die Versorgung von Wunden in der Notaufnahme als defizitär zu betrachten. Bei der ambulanten Wundversorgung kommt zum personellen Aufwand ein hoher Anteil an Materialkosten hinzu. Zusätzlich zu den kalkulierbaren Kosten, bestehen Kostenfaktoren, wie Verwaltungs‑/Aufnahmepersonal, Reinigung, Abrechnung sowie die Zuschläge für Nachtarbeit und anfallenden Freizeitausgleich auf die in der hier vorliegenden Berechnung zur vereinfachten Darstellung verzichtet wurde, die jedoch durch die EBM-Pauschale 02301 in keiner Weise abgedeckt werden. Krankenhäuser haben hohe Vorhaltungskosten, die durch eine oftmals unzureichende Vergütung für die Versorgung von Notfällen zu einer systematischen Unterdeckung der Notaufnahmen führen [21]. Diese Vorhaltekosten werden von Taheri et al. als ein nichtunerheblicher Betrag beschrieben, der durch die Vergütung des EBM, welcher auf die Praxisstruktur ausgerichtet ist, nicht gedeckt werden kann [27].

Ein möglicher Lösungsansatz für die Problematik der Kostenunterdeckung der ambulanten Wundversorgung in der Notaufnahme eines Krankhauses könnte eine Anpassung der Bewertungsmaßstäbe sein. Hier könnte eine Aufwertung für eine Kostendeckung sorgen. Auch eine Zusatzziffer, die Kliniken abrechnen könnten, wenn die Versorgung nicht durch die Vertragsärzte stattfindet, könnte zu einer Kostendeckung beitragen, ohne das EBM-Vergütungssystem für die Vertragsärzte zu tangieren. Unabhängig von der Tatsache, dass gemäß § 75 Abs. 1 Satz 2 SGB V die kassenärztliche Vereinigung verpflichtet ist, die vertragsärztliche Versorgung in den sprechstundenfreien Zeiten in Form eines Notdienstes sicherzustellen, besteht seitens des Versicherten keine zwingende Notwendigkeit, vorrangig diesen Notdienst in Anspruch zu nehmen. Die Patienten haben nach § 76 Abs. 1 SGB V das Recht, in der besonderen Situation eines Notfalls nach freier Wahl auch andere als Vertragsärzte und somit Krankenhäuser zu konsultieren [21]. Hier könnte durch eine konsequentere Patientensteuerung, wie ein Gatekeeper-Modell durch den ärztlichen Bereitschaftsdienst (ÄBD) für eine Entlastung der Krankenhäuser gesorgt werden. In einer Befragung wurde gezeigt, dass in 10 hessischen Notaufnahmen nur 23,57 % der Patienten zuvor Kontakt mit dem ÄBD hatten [1, 21]. Durch die ambulanten Praxen erfolgt jedoch wenige bis keine bildgebende Diagnostik, weshalb fast 15 % der Patienten am selben Tag noch in einer Notaufnahme weiterbehandelt werden. Von diesen Patienten werden 8,8 % stationär aufgenommen [17]. Im ärztlichen Bereitschaftsdienst findet sich ein breites Spektrum an Diagnosen, von denen kumulativ ca. 25 % traumatologisch einzuordnen sind. In den Notaufnahmen machen diese ca. 50 % aus. Haas et al. betrachten ein Gros der unfallchirurgischen Krankheitsbilder aufgrund notwendiger Diagnostik als „krankenhausspezifisch“ [10]. Betrachtet man das Gutachten des Sachverständigenrates (SVR) für Gesundheit aus 2018, wurden hier Defizite in der Patientensteuerung und Fehlanreize der Vergütungssysteme adressiert [20]. Notfallpatienten werden verstärkt stationär aufgenommen, weil die Abrechnung ambulanter Leistungen durch den EBM nicht kostendeckend ist, während bei stationärer Aufnahme über das erheblich lukrativere DRG-System abgerechnet werden kann. Ein sektorenübergreifendes Vergütungssystem und eine bundeseinheitliche Definition von Versorgungsstufen auf Fachabteilungsebene könnten über Multiplikationsfaktoren sowohl Kliniken der Grund- und Regelversorgung als auch Maximalversorgern eine angemessenere Betriebskostenfinanzierung ermöglichen [31]. Auch Biberthaler et al. zeigten, dass in 14 Notfallstationen mit 524.716 Patienten pro Jahr über 43 % der Patienten aufgrund eines Traumas oder einer orthopädischen Erkrankung vorstellig wurden, davon wurden 14 % mit tiefen Wunden behandelt und 21 % am selben Tag aufgenommen. Die Patienten kamen rund um die Uhr und erfordern die ständige Anwesenheit eines Trauma- und Notfallchirurgen [5]. Um dies sicherzustellen, schlägt der SVR eine integrierte Leitstelle (ILS) zur gezielten Koordination der Patienten in der Notfallversorgung rund um die Uhr vor. Patientenanrufe sollen unter einer Rufnummer zusammenlaufen, und von erfahrenen Fachkräften soll eine qualifizierte Ersteinschätzung (Triage), basierend auf leitliniengestützte Notfallalgorithmen, vorgenommen werden. Das interdisziplinäre Notfallzentrum, bestehend aus Notfallambulanz und vertragsärztlicher Bereitschaftsdienstpraxis, ist an ein Krankenhaus angegliedert und wird gemeinsam von kassenärztlichen Vereinigungen (KV) und Krankenhäusern getragen. Die Vergütung soll mit entsprechenden Pauschalen, die u. a. Vorhaltekosten der Häuser berücksichtigen, aus einem extrabudgetären Finanzierungstopf erfolgen [32].

Für die Behandlung von Patienten in Universitätskliniken können gesonderte Verträge mit den Krankenkassen abgeschlossen werden. Die Hochschulambulanzen erhalten von den Krankenkassen pro Behandlungsfall eine Pauschale. Die Vergütung erfolgt nicht aus der Gesamtvergütung über die KV, sondern direkt über die Kassen. Diagnosestellung und leitende Therapieentscheidungen in Hochschulambulanzen sind ausschließlich Fachärzten vorbehalten. Die Aufrechterhaltung einer Hochschulambulanz ist jedoch im Notdienst schwierig, da ein Großteil der Notdienste im Vordergrund durch Assistenzärzte geleistet wird [2].

Als Limitation dieser Studie bleibt aufzuzeigen, dass im komplexen Umfeld einer Klinik nicht alle Faktoren erfasst werden können. Wie bereits zuvor angemerkt, sind die Kosten für Verwaltungs‑/Aufnahmepersonal, Reinigung, Abrechnung sowie die Zuschläge für Nachtarbeit und anfallenden Freizeitausgleich nicht erfasst worden. Ebenso sind die Vorhaltekosten für Personal und Investitionskosten nicht berücksichtigt. Ausgegangen wurde vom Regelfall einer Wundversorgung; eine mögliche Beteiligung weiterer Ärzte oder Pflege aufgrund komplexerer Versorgung wurde nicht berechnet. Die Minderdeckung der Wundversorgung wurde bereits ohne die zuvor genannten Faktoren gezeigt, welche letztendlich zu einem noch drastischeren Defizit führen würden. Somit ist davon auszugehen, dass die Problematik auch auf jede kleinere Klinik zutrifft.

Die derzeitigen Erlöse der Notfallwundversorgung im komplexen Klinikumfeld spiegeln nicht den finanziellen Aufwand wider. Für die Kliniken bedeutet das hohe jährliche Fehlbeträge, welche die Finanzierbarkeit der Notaufnahmen auch weiterhin erschweren. Unter anderem können so eine ausreichende personelle Ausstattung sowie die damit verbundene Behandlungsqualität und die Verkürzung von Wartezeiten für Patienten nicht gegenfinanziert werden.

## Fazit

Die bisherige Abrechnung der ambulanten Wundversorgung in den Notaufnahmen der Krankhäuser durch den EBM ist höchst unzureichend. Eine Alternative oder Erweiterung des Vergütungssystems für den Krankhaussektor bzw. die Einführung einer konsequenteren Patientensteuerung scheinen unumgänglich, um der defizitären Finanzstruktur der Notaufnahmen entgegenzuwirken.
